# Gypsum and carbon amendments influence carbon fractions in two soils in Ohio, USA

**DOI:** 10.1371/journal.pone.0283722

**Published:** 2023-04-04

**Authors:** Maninder K. Walia, Warren A. Dick

**Affiliations:** The Ohio Agricultural Research and Development Center, The Ohio State University, Wooster, Ohio, United States of America; Assam University, INDIA

## Abstract

Carbon sequestration as influenced by management practices such as soil amendments is not yet fully understood. Gypsum and crop residues can improve soil properties, but few studies have focused on their combined effect on soil C fractions. The objective of this greenhouse study was to determine how treatments affected different forms of C, i.e., total C, permanganate oxidizable C (POXC), and inorganic C in 5 soil layers (0–2, 2–4, 4–10, 10–25, and 25–40 cm). Treatments were glucose (4.5 Mg ha^-1^), crop residues (13.4 Mg ha^-1^), gypsum (26.9 Mg ha^-1^) and an untreated control. Treatments were applied to two contrasting soil types in Ohio (USA)—Wooster silt loam and Hoytville clay loam. The C measurements were made one year after the treatment applications. Total C and POXC contents were significantly higher in Hoytville soil as compared to Wooster soil (P < 0.05). Across both Wooster and Hoytville soils, the addition of glucose increased total C significantly by 7.2% and 5.9% only in the top 2 cm and 4 cm layers of soil, respectively, compared to the control treatment, and residue additions increased total C from 6.3–9.0% in various soil layers to a depth of 25 cm. Gypsum addition did not affect total C concentrations significantly. Glucose addition resulted in a significant increase in calcium carbonate equivalent concentrations in the top 10 cm of Hoytville soil only, and gypsum addition significantly (P < 0.10) increased inorganic C, as calcium carbonate equivalent, in the lowest layer of the Hoytville soil by 32% compared to the control. The combination of glucose and gypsum increased inorganic C levels in Hoytville soils by creating sufficient amounts of CO_2_ that then reacted with Ca within the soil profile. This increase in inorganic C represents an additional way C can be sequestered in soil.

## Introduction

Soil C, as the third-largest C pool in the Earth system, plays a vital role in the global C cycle and in affecting potential climate change [1]. More than two to three times as much organic C is held in soils worldwide as terrestrial biomass [2]. On a global scale, the estimated size of soil organic C (SOC) to a 1-m depth is 1500 to 1600 Pg [3–5]. Therefore, soil organic matter is regarded as a potentially important C sink that can help mitigate climate change by sequestering C removed from the atmosphere by plants [6].

Soil inorganic C (SIC) in arid and semi-arid regions is primarily in the form of carbonates and represents more than 90% of the total C in these soils [7, 8]. On a global scale, the SIC pool’s estimated size is about 750 Pg to 1 m depth [5]. The major inorganic C mineral phases are calcite and dolomite. Calcite has a considerably higher dissolution rate than dolomite, approximately 100-fold [9].

The movement of C between the soil and the atmosphere is bidirectional, thus making the soil C reservoir truly dynamic [10]. Consequently, C storage in soils reflects the balance between the opposing processes of accumulation and loss. Carbon flux between the atmosphere and terrestrial ecosystems involves both SOC and SIC.

Changes in soil C storage, i.e., sequestration, occur as a result of various management practices. For example, the C flux between SOC pools and the atmosphere depends on biomass production, organic materials input, and soil respiration [11, 12]. The SIC pools exchange C with the atmosphere through a series of physical and chemical reactions such as C sequestration by carbonate formation or CO_2_ release by acidification and leaching [1, 13, 14]. Practices that favor an accumulation of carbonates in the soil include efficient irrigation with surface waters in arid and semi-arid regions (leaching less than 30% of the applied water), irrigation with groundwaters at elevated CO_2_ concentrations, application of gypsum to alkaline soils, and use of nitrate fertilizers [8].

Land-use changes or agricultural management practices can lead to changes in total soil C content. However, these changes often occur gradually and against the largest C pool in the terrestrial biosphere. Thus, minor/small soil C stock changes are often difficult to detect in the short or medium-term [15, 16]. Moreover, considering bulk total C as one homogeneous pool ignores the variation in relative proportions of various C fractions [17, 18].

Soil organic C is commonly measured by dry combustion with automated analyzers [19] or by the less sensitive wet chemical oxidation method [20]. The analysis of total C via dry combustion is regarded as the standard method for measuring C in soil due to its high precision and accuracy [21–23]. Changes in small but relatively labile fractions of soil organic C (SOC) may be used to provide an early indication of soil degradation or improvement in response to management practices [18]. The labile or active fractions of soil C contrasts with highly recalcitrant/passive pools that are only slowly altered by microbial activities [18]. Active C can be measured as permanganate oxidizable C (POXC) using 0.02 mol L^-1^ KMnO_4_ solution. This method is rapid, inexpensive, and can be modified for use under field situations [18, 24]. Weil et al. [18] showed that POXC was related to most soil microbial activity measures, including microbial biomass carbon, substrate-induced respiration, soluble carbohydrate C, and total SOC. Other studies have found significant positive relationships between POXC and microbial biomass [25–28].

Soil inorganic C, e.g., carbonates, can be determined in soil by several methods [9]. Commonly, they are determined by measuring CO_2_ production after the addition of hydrochloric (HCl) acid into the soil [29]. This is a simple and inexpensive method. Dreimanis [30] demonstrated the quantitative method of determining calcite, dolomite, and the total percentage of carbonates by the Chittick apparatus, which is rapid, simple, inexpensive, and reasonably accurate. The Chittick apparatus is designed primarily to determine the volume of CO_2_ that evolved from carbonates reacting with acid.

For maintenance and improvement of soil properties for continuous agricultural production on a long-term basis, it is crucial to managing the soil properly. Organic amendments are a viable means of improving the productivity of the soils [31]. A major source of organic input to the soil is the large amounts of crop residues generated, approximately 1.5 billion metric tons per year in the United States and 4 billion metric tons per year globally [32]. Application of crop residues increases soil organic C content [33, 34]. Crop residues are also a valuable source of macronutrients (e.g., N, P, K, Ca, Mg) and micronutrients (e.g., Fe, Zn, and Mn) essential for plant growth and development. Crop residues returned to the soil can improve soil quality and productivity through favorable effects on soil’s physical properties [35].

Gypsum is another material that is often used as a soil amendment. Application of gypsum (calcium sulfate dihydrate: CaSO_4_. 2H_2_O) to agricultural soils has been practiced for more than 250 years and is one of the earliest forms of fertilizers used in the United States [36]. The positive effects of gypsum have been observed in diverse soil and weather conditions [37–39]. FGD gypsum, like other sources of gypsum, has the potential to improve soil quality when applied to agricultural land. This can indirectly increase agricultural productivity [40, 41]. Several reviews have been published that summarize the effects of gypsum on soil/plant systems [42–45].

For studies where a quick response to added C is desired, glucose is often used as a stand-in for readily available C. Glucose decomposes quickly and is known to improve the soil physical properties such as aggregation [46] and biological properties such as microbial biomass C, enzyme activities, microbial growth, etc. [47, 48].

Global change has altered temperature, precipitation, N availability, and many other environmental factors [49, 50], and these changes are likely to have a great impact on soil C. An accurate quantification of soil organic C and soil inorganic C stocks are of fundamental interest to more accurately predict future soil C dynamics. Recent studies have documented the importance of crop residue, readily available C (i.e., glucose), and gypsum additions to soil on its physical and chemical properties [46, 51]. However, little is known about the combined effects of crop residue, glucose, and gypsum on total soil C and its fractions. The objective of this study was to determine the effects of the increasingly used farm practice of applying gypsum to the soil and how this application interacts with C sources (i.e., crop residues and glucose) to impact total C, oxidizable C, and inorganic C in two soils in Ohio and, potential C sequestration in soil.

## Materials and methods

## Experimental setup

A greenhouse experiment was conducted at The Ohio Agricultural Research and Development Centre of The Ohio State University, Wooster, Ohio, USA. A Wooster silt loam soil (fine-loamy, mixed, mesic Typic Fragiudalfs) and a Hoytville clay loam soil (fine, illitic, mesic Mollic Epiaqualfs) were collected from the top 30 cm as two contrasting soils from university farms located near Wooster and Custar, Ohio, USA, respectively in the summer of 2013. Each soil type was screened through a 2-mm mesh sieve and then mixed thoroughly before selected soil properties were measured before the experiment initiation (Table 1) [46, 51, 52]. Soil properties measured were soil pH using a 1:1 ratio of soil: deionized water [53], texture using hydrometer method [54], total C and N using high-temperature combustion [19], Bray P-1 [55], and exchangeable bases (Ca, K, and Mg) with 1 M NH_4_OAC [56].

**Table 1:** Soil chemical characteristics (Adapted from [4,6, 51]).

	Soil	
Parameter	Wooster	Hoytville
pH	6.15	6.80
Texture Classification	Silt loam	Clay loam
Sand (%)	16	22
Silt (%)	59	30
Clay (%)	25	48
Carbon (%)	0.72	2.14
Nitrogen (%)	0.09	0.24
Bray P-1 (mg/kg)	6.5	22.2
NH_4_OAc Extracted (mg/kg)		
Ca^2+^	1140	2730
Mg^2+^	184	365
K^+^	72.9	254
CEC^a^ (cmol(+)/kg)	7.4	19.2

^a^ CEC = cation exchange capacity.

The materials applied as soil amendments were crop (corn) residues, gypsum, and glucose. Corn residues were collected from the field and dried in a drying oven at 60°C before being ground to pass a 2-mm mesh sieve. The other two soil amendments, glucose, and gypsum were obtained from Fisher Scientific. Soil amendments were analyzed for total C and N (high-temperature combustion), and total P, K, Ca, Mg, S, B, Fe, Mo, Na, and Zn concentrations (microwave-assisted nitric acid digestion followed by inductively coupled plasma–atomic emission spectrometry (ICP–AES) measurement) (USEPA 3051) (Table 2). More details on the experimental material and fertility of soil have been reported previously [51, 52, 57].

**Table 2:** Concentration of elements in the inputs added to the soil (Adapted from [46, 51]).

Element	Crop (Corn) Residues		Glucose	Gypsum	
	---------------------------------------------- % -----------------------------------------				
N	0.90	ND^a^			ND
C	45.0	39.9			ND
	--------------------------------------------mg kg-1 -----------------------------------				
P	654	<8.32			<8.32
K	8560	<90.3			223
Ca	2922	<15.5			196,735
Mg	1915	<1.65			6.58
S	971	<13.0			154,450
B	0.99	<0.35			14.3
Fe	425	29.8			1.00
Mo	0.48	0.73			1.13
Na	25.7	<20.9			52.0
Zn	41.5	<0.78			<0.78

^a^ND, not determined.

Soil columns 60 cm long and 20 cm in diameter were made from polyvinyl chloride pipe as described in Walia and Dick [51]. Each soil type was thoroughly mixed to provide a uniform material. The soil columns were filled first with a layer of gravel to a depth of 5 cm. The homogenized soil was placed to a depth of 35 cm over the gravel, except for the control/untreated columns that were filled to a depth of 55 cm. The columns were filled manually and compacted against gravity. The top 20 cm layer of the treated columns consisted of soil treated with amendments, i.e., corn residues (0 and 13.4 Mg ha^-1^), gypsum (0 and 26.9 Mg ha^-1^), and glucose (0 and 4.5 Mg ha^-1^), and their combinations. The corn residues were mixed into the soil at the time the columns were filled with soil. The gypsum was surface applied in four equal applications of 6.7 Mg ha^-1^_._ The first application was made at the time the columns were filled with soil. The other three applications of gypsum were applied on the soil surface one, three, and five months after ryegrass was sown. Similarly, glucose was initially surface applied at a rate of 2.25 Mg ha^−1^ at the time columns were filled. A second equal application of glucose was made onto soil surface three months after ryegrass was seeded. A control treatment with no soil amendments was included in the experimental design. There were four replicates of each treatment or treatment combination, which yielded a total of 64 experimental units. The experiment design was a complete block factorial.

The soil columns were seeded with perennial ryegrass (*Lolium perenne*) in September at the rate of 27 kg ha^-1^. The perennial ryegrass was chosen due to its rapid growth and fibrous root system. The P and K fertilizers as single super phosphate and muriate of potash, were applied at rates of 25 and 75 kg ha^−1^, respectively, at planting. However, nitrogen as urea was surface applied in three equal doses (56 kg ha^-1^ each) two, three, and four months after seeding. The rates of seeding and fertilizer were chosen based upon NRCS-Ohio recommendations for grasses grown in Ohio. All soil columns received an equal amount of water, based upon plant needs until the end of the experiment, to ensure that the only variable factors were the soil amendments. The greenhouse temperature was maintained at 22°C/18°C (day/night) throughout the experiment period.

### Soil sampling and methods

Soil materials were collected at the end of the experiment i.e., one year after the addition of amendments to the soil, by cutting the soil columns (diameter 20 cm) into layers of thickness 0–2, 2–4, 4–10, 10–25, and 25–40 cm. More complete experimental details along with a timeline are provided elsewhere [46, 51, 52, 57]. This paper reports measurement on the carbon fractions in soils. Collected soil was air-dried and sieved by hand through a 2-mm mesh sieve. Total soil C was determined by the dry combustion method using Elementar America’s VarioMax C-N combustion analyzer. Permanganate-oxidizable C was measured based on Weil et al. [18] by using a spectrophotometer. A detailed protocol of this method can be found at http://lter.kbs.msu.edu/protocols/133. Calcium carbonate equivalents (CCE) in soil samples were determined by the gasometric method of Dreimanis [30], employing a Chittick apparatus. In this method, two readings of the volume of the evolved CO_2_ were taken. First, for calcite, after the soil sample had completely mixed with the hydrochloric acid (HCl) and usually after 30 seconds. Second, for dolomite, a reading was taken 30 minutes after the beginning of analysis when the volume of CO_2_ evolved no longer changes. Calcium carbonate equivalent is determined from values obtained for calcite and dolomite (on a weight basis) according to the following formula:

%CCE=Calcite(%)+(1.0847*Dolomite(%))

Total soil C stock for the entire profile 0–40 cm was also calculated for each treatment. Soil C concentrations (mg g^-1^ dry soil) was multiplied by mean bulk density reported elsewhere [46] and respective soil depth of each layer (2, 2, 6, 15, and 15 cm) to convert total C concentration to mass per area basis (Mg ha^-1^) for all treatments.

### Statistical analysis

Data were analyzed using the general linear model (GLM) with Tukey’s multiple comparisons [58]. Fixed factors were glucose, gypsum, residue, soil, and their two-way interaction. Replication was considered as a random factor. Treatment mean comparisons were made using Tukey’s HSD at P < 0.10.

## Results and discussion

### Total C

Total C in soil, prior to treatment applications, was significantly higher in the Hoytville clay loam, approximately three times greater than the Wooster silt loam (Table 1). Perennial ryegrass production in these two soils, as above-ground biomass, was also significantly higher in the Hoytville soil (44.3 g pot^-1^) as compared to the Wooster soil (25.9 g pot^-1^) [51]. Roots provide a source of organic C that tends to be mixed into the soil after they die. Also, when roots are alive, they release organic compounds rich in C that serve as energy sources for microbiota [59] as well as CO_2_ via respiration, which can also impact C fractions in soil.

**Fig 1:**
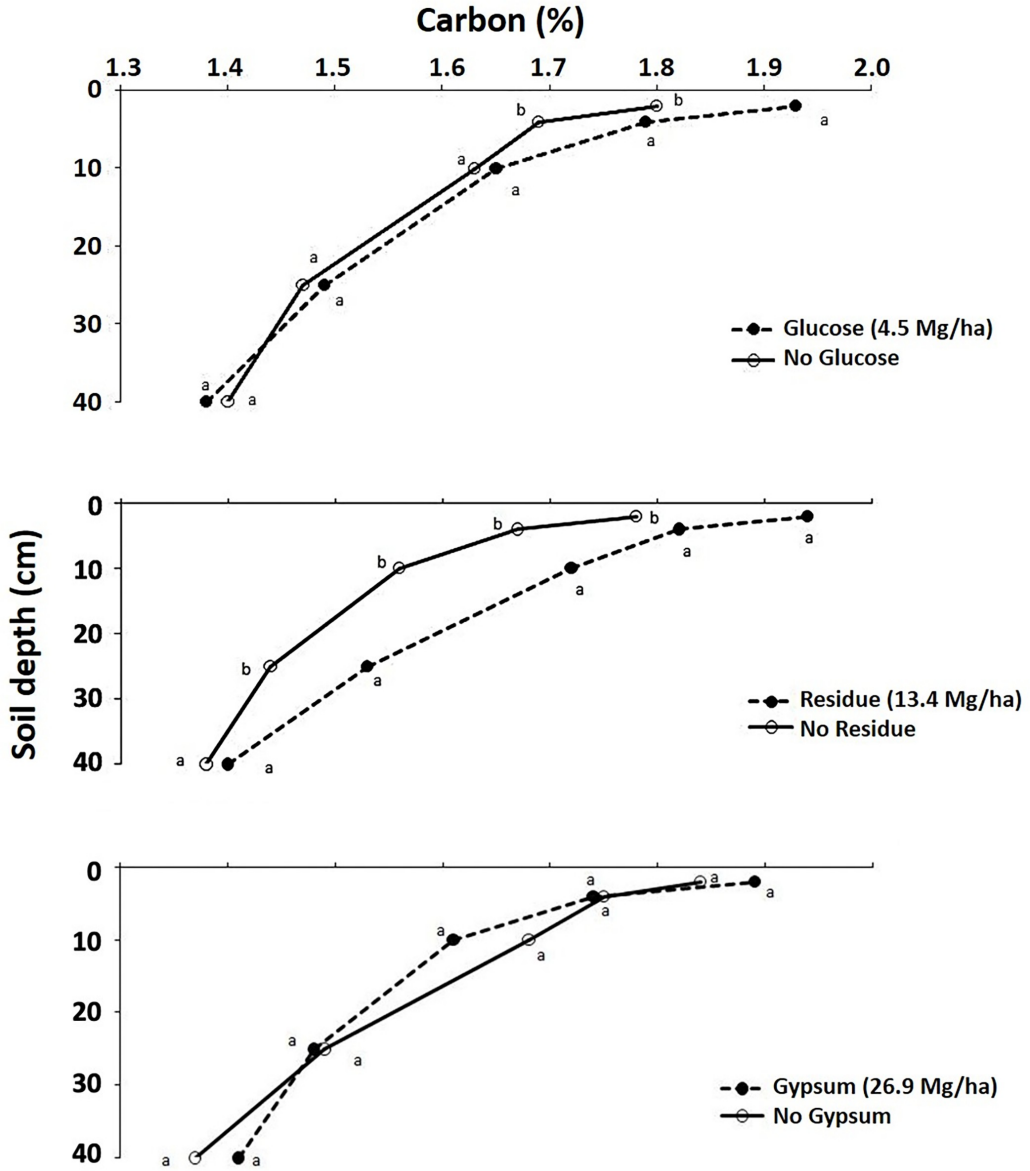
Effect of residue, glucose and gypsum on % carbon concentrations in different soil layers averaged across the Wooster and Hoytville soils. Means with the same letter within the same layer are not statistically different (P < 0.10).

Total C content in soils with different treatments varied from 1.37–1.94% (Fig 1). The addition of glucose averaged acrpss the two soils resulted in a significant increase in total C, but only in the 0–2 and 2–4 cm soil layers by 7.2 and 5.9%, respectively, compared to the control (Table 3 and Fig 1). Glucose is a polysaccharide that is a quickly decomposable material and an excellent source of C. It exerts a rapid transient stabilization effect on aggregates [46, 60].

**Table 3:** Probability values of significant factors of direct effects and interaction effects on total soil C concentrations in different soil layers averaged across the two soils.

Factors	0–2	2–4	4–10	10–25	25–40
Glucose	**0.073** ^a^	**0.072**	0.793	0.739	0.356
Residue	**0.035**	**0.008**	**0.005**	**0.056**	0.541
Gypsum	0.453	0.857	0.145	0.765	0.202
Soil	**<0.001**	**<0.001**	**<0.001**	**<0.001**	**<0.001**
Glucose*Residue	0.132	0.181	0.354	0.206	0.566
Glucose*Gypsum	0.443	0.472	0.464	0.995	0.111
Glucose*Soil	0.564	0.670	0.303	0.701	0.150
Residue*Gypsum	**0.029**	0.151	0.491	0.591	0.280
Residue*Soil	0.309	0.169	0.416	0.230	**0.091**
Gypsum*Soil	**0.058**	0.110	0.170	0.703	**0.031**

^a^Bold values denote statistical significance at the P < 0.10 level of significance.

A significant increase was also observed, when averaged across the two soils, in total C content with the addition of crop residues in soil layers, i.e., the increases in the 0–2, 2–4, 4–10, and 10–25 cm soil layers were 9.0, 8.4, 9.5, 6.3%, respectively, as compared to the control (P < 0.10) (Table 3 and Fig 1). Many studies have shown that crop residues returned to the land can increase or maintain SOC content [33, 34, 61]. Duikar and Lal [62] observed that the addition of residue for seven years had a positive effect on soil organic C content in the 0–10 cm soil layer but not in 10–30 cm. Walia et al. [63] observed that the addition of crop residues along with fertilizers increased organic C content as compared to a control in the top 30 cm of soil. The interaction of residue with gypsum was found to be significant only in the 0–2 soil layer for total C (Table 3).

Total C stock after treatments was significantly affected by soil types, being greater in the Hoytville soil (112 Mg ha^-1^) as compared to Wooster silt loam soil (36 Mg ha^-1^). This was attributed to higher clay and carbon content in the Hoytville soil. Aggregation and SOC concentration represents the integrative effects of soil type, environment, plant species, and soil management practices [64, 65]. Total C stocks in the 0–40 cm soil profile did not vary significantly with the alone addition of glucose, residue, or gypsum compared to untreated control. However, the interaction of residue with gypsum was found to be significant for increasing total C stock (P < 0.10). The combined application of residue and gypsum increased total C content (76.0 Mg ha^-1^) to the highest level compared to other treatments. The least amount of total C was found in soil with residue application only (71.6 Mg ha^-1^), which was attributed to the residues stimulating more microbial activity and thus causing a priming degradation effect of the native soil organic matter as well as the added organic matter as a residue [66]. As noted above, the greatest amount of total C (76.0 Mg ha^-1^) was found when both gypsum and residue were added together to the soil. This is due to the abundance of Ca^2+^ from the gypsum application, which forms clay-Ca^2+^-organic matter interactions and physically protects the added residue C from degradation [46]. This was also supported by the findings that total C (73.3 Mg ha^-1^) in the soil was greater when gypsum alone was added to soil compared to only residue addition (71.6 Mg ha^-1^). However, the untreated control held total C almost similar to the combined application of gypsum and residue (75.5 Mg ha^-1^). This may initially seem to be an unlikely finding but was attributed to the high rate of gypsum application inhibiting microbial respiration and improved physical protection of the C in the soil. Earlier studies [67–69] also reported lower microbial respiration in Ca-rich environments. Also, Minick et al. [70] observed a negative correlation between Ca_Exch_ concentration and SOC leaching losses. Thus, the present study’s findings suggest that gypsum may be a useful soil amendment that will aid in C sequestration in the soil, mainly when applied in combination with C amendments such as crop residues.

### Permanganate oxidizable C (POXC)

Permanganate oxidizable C was significantly correlated with total soil C (R = 0.42, P<0.0001). A strong positive relationship between POXC and SOC has been reported previously [18, 71, 72]. POXC made up only 4.9% of the total soil C, with mean values of 805 mg POCX kg^-1^ soil (0.081%) compared to 1.63% of total soil C. Similar results have been reported by Culman et al. [73] that POXC comprised only 4% of the total SOC when measured across a wide range of soil types, ecosystems, and geographic areas. The large differences between SOC and POXC are due to the fact that total SOC relies on complete oxidation of all soil C, while POXC relies on partial oxidation of the C pool [69]. However, the C that is partially oxidized and measured by POXC is considered highly active in soil [74].

**Fig 2:**
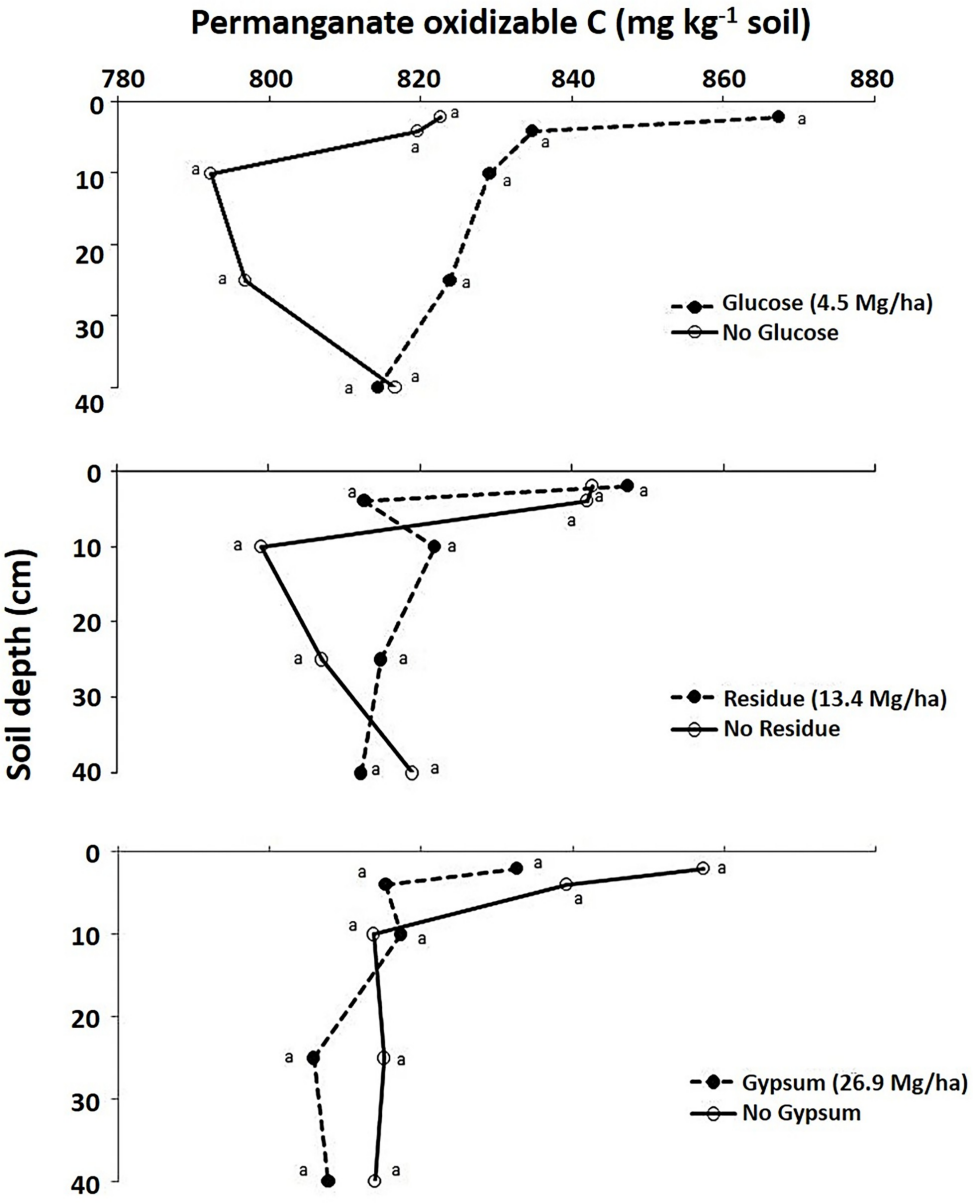
Effect of residue, glucose and gypsum on POXC in different soil layers averaged across the Wooster and Hoytville soils. Means with the same letter within the same layer are not statistically different (P<0.10).

Similar to the total C content, POXC was significantly higher in the Hoytville soil as compared to the Wooster in all soil layers (P<0.10). The addition of glucose, when averaged across the two soils, increased POXC in all soil depths but not significantly compared to no addition of glucose (Fig 2). This was attributed to the long lag time between glucose addition and POXC measurements. The addition of residue did not significantly increase POXC in various soil layers. Likewise, gypsum addition did not significantly influence POXC compared to the untreated control (Fig 2).

### Inorganic C (Calcite, dolomite, and calcium carbonate equivalent)

The Wooster soil samples treated with glucose, residue, and gypsum, and control were analyzed for concentrations of inorganic C fractions by the Chittick apparatus. However, no significant differences were observed between these treatments.

**Fig 3:**
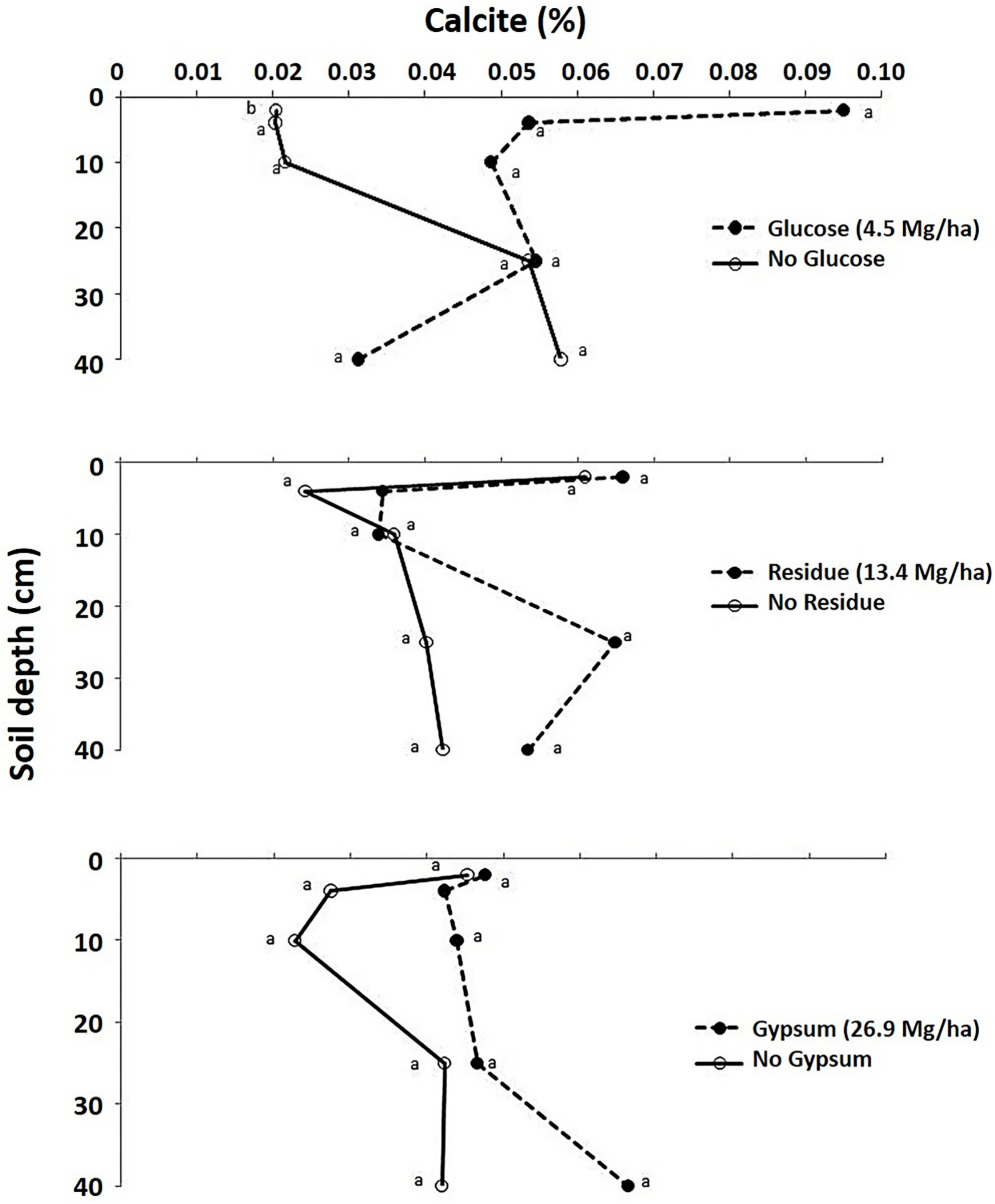
Effect of glucose, residue and gypsum addition on calcite (%) in different Hoytville soil layers. Means with the same letter within same layer are not statistically different (P<0.10).

For Hoytville soils, however, the addition of glucose alone resulted in a significant increase in calcite concentration by 380% in the 0–2 cm soil layer as compared to untreated control (Table 4; Fig 3). Calcite is CaCO_3,_ with 40% Ca. This was attributed to an abundance of CO_2_ in soil from the decomposition of glucose and a soil pH value of 6.65 at the surface layer only [51]. However, no significant differences were observed in calcite concentrations below 2 cm due to a significant decrease in soil pH with glucose in lower layers resulted from acidification upon glucose metabolism [51]. Glucose metabolism results in the formation of some organic acids [75, 76].

**Table 4:** Probability values of significant factors on inorganic C fractions in Hoytville soils in different soil layers.

	---------------------------------Soil layer (cm) ----------------------------------								
Factors	0–2	2–4		4–10		10–25		25–40	
	**Calcite**								
Glucose	**0.100** ^a^		0.419		0.316		0.975		0.367
Glucose*Gypsum	**0.100**		0.455		0.907		**0.091**		0.429
	**Dolomite**								
Glucose	**0.082**		**0.014**		**0.037**		0.818		0.398
Glucose*Gypsum	**0.079**		**0.009**		0.376		**0.090**		0.301
**Calcium carbonate equivalent (CCE)**									
Glucose	**0.072**		**0.013**		**0.033**		0.816		0.439
Gypsum	0.590		0.851		0.652		0.679		**0.100**
Glucose*Gypsum	**0.081**		**0.014**		0.308		**0.067**		0.330

^a^Bold values denote statistical significance at the P < 0.10 level of significance.

**Fig 4:**
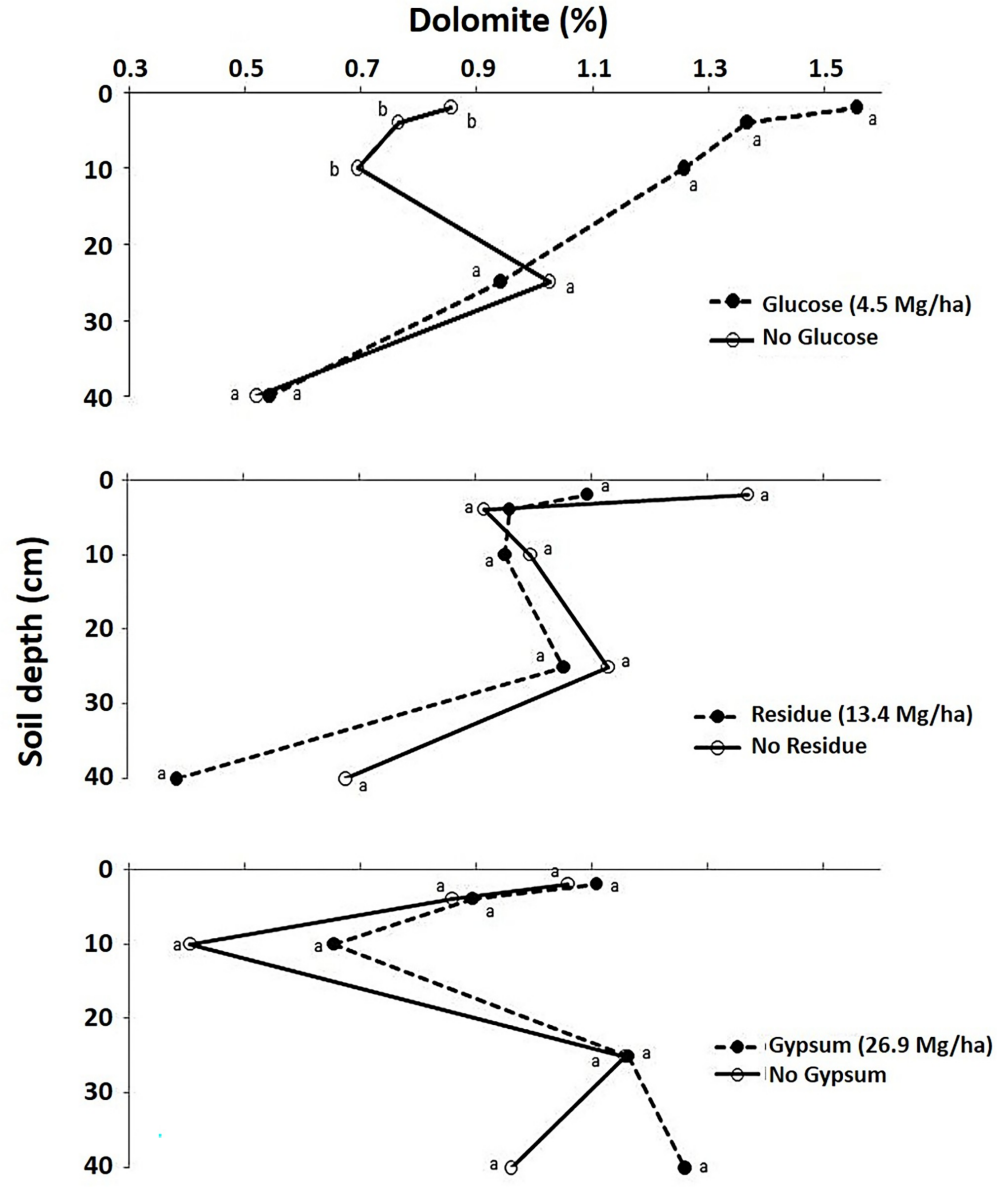
Effect of glucose, residue and gypsum addition on dolomite (%) in different Hoytville soil layers. Means with the same letter within same layer are not statistically different (P<0.10).

**Fig 5:**
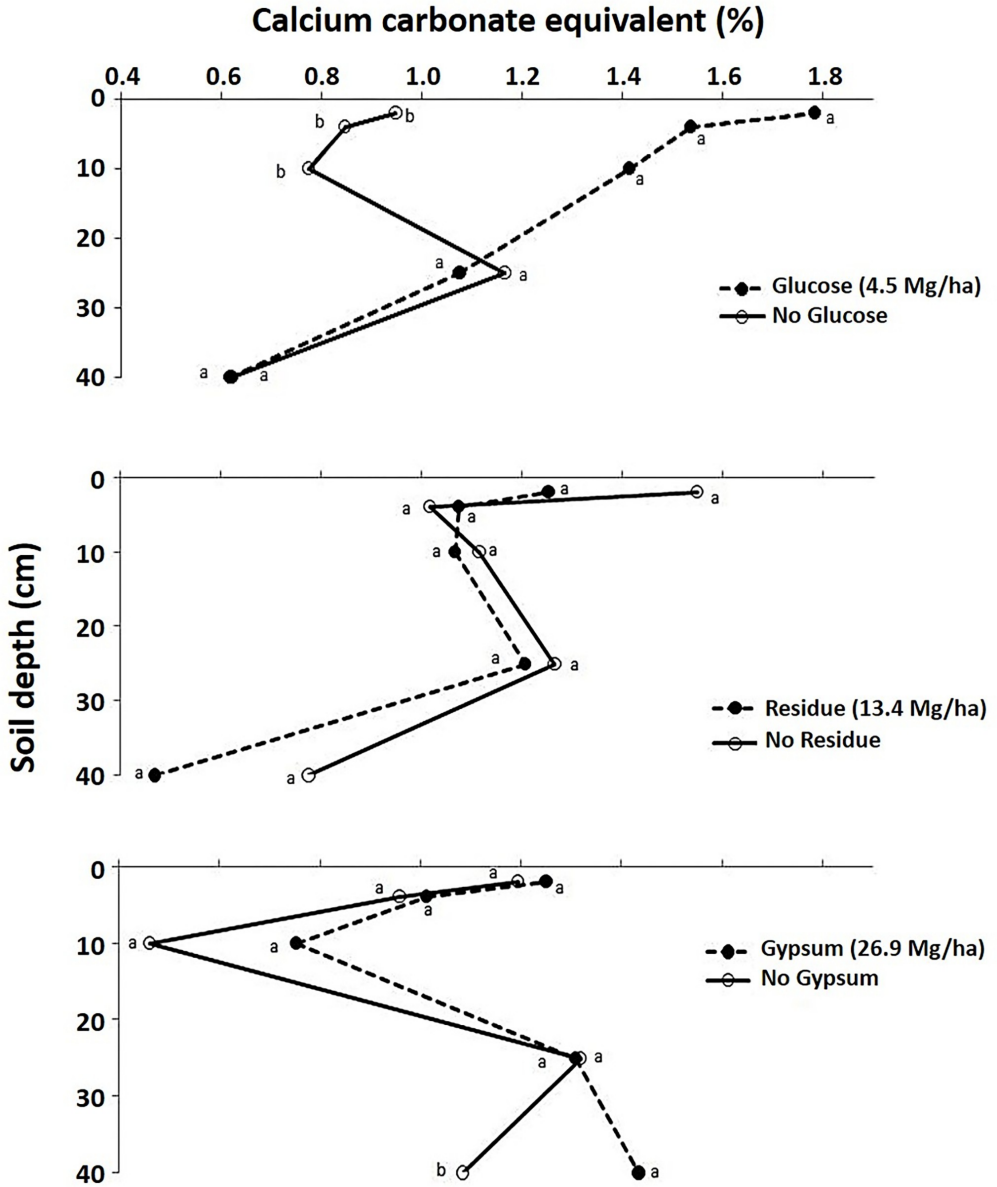
Effect of glucose, residue and gypsum addition on calcium carbonate equivalent concentrations (%) in different Hoytville soil layers. Means with the same letter within same layer are not statistically different (P<0.10).

Dolomite [CaMg(CO_3_)_2_ with 22% Ca and 13% Mg] concentrations were also significantly increased with glucose in the top 10 cm, i.e., the 0–2, 2–4, and 4–10 cm Hoytville soil layers by 100, 60, and 70%, respectively, as compared to the control (P < 0.10) (Table 4 and Fig 4). Concentrations of total calcium carbonate equivalents (calculated from values of calcite and dolomite) were also increased significantly in the 0–10 cm soil depths by 100, 61, and 83% in the 0–2, 2–4, and 4–10 cm soil layers, respectively, as compared to the control (Table 4 and Fig 5).

The presence of carbonates is usually associated with neutral to alkaline soils, but solid-phase carbonates in the form of nodules exist in some acid environments [9]. The increase in inorganic C with glucose addition was attributed to increased CO_2_ concentration within the soil profile upon decomposition of glucose. This moved the carbonate reaction forward (Eq 1) due to the CO_2_ reacting with water to form bicarbonate. In the presence of Ca and Mg ions in the soil, calcite and dolomite can then form.


$$CO_{2}\;(g)+H_{2}O‐‐‐>H_{2}CO_{3}\;(aq.)$$ (Eq 1)

$$H_{2}CO_{3}\;(aq.)+2OH^{‐}\;(aq.)‐‐‐>CO_{3}{}^{2‐}(aq.)+4H_{2}O$$ (Eq 2)

$$Ca^{2+}(aq.)+CO_{3}{}^{2‐}‐‐‐>CaCO_{3}\;(s)$$ (Eq 3)

$$Overall\;reaction\;Ca^{2+}+CO_{2}+2OH^{‐}‐‐‐>CaCO_{3}+H_{2}O$$ (Eq 4)

$$Ca^{2+}(aq.)+Mg^{2+}(aq.)+2CO_{3}{}^{2‐}‐‐‐>CaMg{\left(CO_{3}\right)}_{2}\;(s)$$ (Eq 5)

$$\begin{array}{l} \mathrm{Overall}\;\mathrm{reaction}\\ Ca^{2+}+Mg^{2+}+2CO_{2}+4OH^{‐}‐‐‐>CaMg{\left(CO_{3}\right)}_{2}+2H_{2}O \end{array}$$ (Eq 6)

The addition of residue increased the calcite concentration in all depths of the Hoytville soil but not significantly (Fig 3). Residues can release basic cations during decomposition, and these basic cations can react with CO_2_ [77, 78]. Moreover, the release of CO_2_ upon decomposition of residue also moved the calcite forming reaction forward and increased calcite concentrations compared to the control (Eqs 1-6). Dolomite and calcium carbonate equivalent concentrations, however, were not affected by the residue treatment (Figs 4 and 5).

Gypsum addition increased calcite and dolomite concentration compared to control, but no significant effects were observed (Figs 3 and 4). Calcium carbonate equivalent also increased with gypsum, but a significant increase was observed only in one Hoytville soil layer, i.e., the 25–40 cm layer (Table 4 and Fig 5). This increase was 32% due to high pH as compared to the control [51] and also due to the downward movement of Ca applied from gypsum [79–81] and higher concentrations of CO_2_ in the deeper soil layers [82]. Interaction of gypsum with glucose was significant for calcite concentrations in the 0–2 and 10–25 cm soil layers. The addition of glucose and gypsum either alone or in combination increases calcite concentrations compared to control, which was attributed to increased Ca concentrations from gypsum and CO_2_ from readily available C source i.e., glucose decomposition. Gypsum with glucose interaction was significant for dolomite and calcium carbonate equivalent concentrations in the 0–2, 2–4, and 10–25 cm soil layers. The addition of glucose or gypsum either alone or in combination increases dolomite and calcium carbonate equivalent content compared to the control. These significant interactions were attributed to both pH and also an abundant supply of CO_2_ and Ca within the soil profile. The addition of Ca^2+^ as gypsum is known to drive a reaction with CO_2_ produced from root respiration, thus producing carbonates (Eqs 1-6). Han and Tokunaga [83] reported that the addition of CaSO_4_ minerals to slightly alkaline soils increases the rate of secondary carbonate formation by depressing the microbial respiration, thereby diminishing the production of soil CO_2_ and its diffusive loss to the atmosphere. However, in lower pH urban soils having artificially enhanced Ca contents, calcium mineralization may occur without further treatment with Ca [84]. A significant increase in inorganic C was also observed with long-term gypsum addition for the past 12 years in Celina soils in Ohio at the soil depth of 60–75 cm only [85] (Tirado-Corbala, 2013). This suggests that inorganic carbonates can also be formed in the soil provided there is a sufficient increase in CO_2_ and Ca^2+^ concentration within the soil profile through the addition of different amendments rich in C and Ca. Furthermore, Mg that was released from soil reacted with increased CO_2_ levels through glucose addition and Ca from gypsum addition, resulting in increased dolomite levels.

## Conclusions

This study demonstrates that the concentration of total C was markedly improved with the addition of glucose and residue to soil depths of 4 and 25 cm, respectively. Gypsum alone addition did not cause a significant increase in the total C content. However, gypsum addition in combination with residue increased total C stock in the soil by improving physical protection of the added C. Gypsum also increased inorganic C in the lowest soil layer (e.g., 25–40 cm) in the Hoytville soil, and this was attributed to the reaction of CO_2_ concentrations, that is often enriched in lower soil profile layers, with the abundant Ca concentrations within the soil profile through the Ca-rich gypsum amendment. Similarly, glucose being a readily available source of C, also increased inorganic forms of C significantly in topsoil layers in the Hoytville soil, presumably by greatly increasing CO_2_ levels that then reacted with Ca and Mg already present in the soil. This study suggested that the addition of gypsum as an amendment in conjunction with inputs of C has the potential of increasing soil organic C levels in soil via physical protection mechanisms. Inorganic C levels in soil may also be increased in subsoils by the addition of Ca as gypsum, and this represents an additional way of C sequestration and thus reduce the C loss to the atmosphere.

## Supporting information

S1 Table Responses of permanganate oxidizable C, calcite, dolomite and calcium carbonate equivalents to experimental treatments of glucose, corn plant residues and gypsum. (PDF)
