# Supplementary material for: Gypsum and carbon amendments influence carbon fractions in two soils in Ohio, USA
Source: PLoS One. 2023 Apr 4;18(4):e0283722. doi: 10.1371/journal.pone.0283722 (PMC10072480; doi:10.1371/journal.pone.0283722)
Supplement: S1 Table — (PDF) [file pone.0283722.s001.pdf]

S1 Table - Responses of permanganate oxidizable C, calcite, dolomite and calcium carbonate equivalents to experimental treatments of glucose, corn plant residues and gypsum.

Permanganate oxidizable C (mg/kg soil)

	Soil depth (cm)				
	2	4	10	25	40
Glucose, 4.5 Mg/ha	867.4	834.9	829.2	824	814.4
No glucose	822.7	819.7	792.4	796.9	816.7

	Soil depth (cm)				
	2	4	10	25	40
Corn Residues, 13.4 Mg/ha	847.4	812.6	821.9	814.7	812.2
No residue	842.7	842	799	807	818.9

	Soil depth (cm)				
	2	4	10	25	40
Gypsum, 26.9 Mg/ha	832.7	815.4	817.4	805.8	807.8
No gypsum	857.3	839.2	813.7	815.1	813.9

Calcite (%)

	Soil depth (cm)				
	2	4	10	25	40
Glucose, 4.5 Mg/ha	0.095	0.054	0.049	0.055	0.031
No glucose	0.020	0.020	0.022	0.054	0.058

	Soil depth (cm)				
	2	4	10	25	40
Corn Residues, 13.4 Mg/ha	0.066	0.034	0.034	0.065	0.053
No residue	0.061	0.024	0.036	0.040	0.042

	Soil depth (cm)				
	2	4	10	25	40
Gypsum, 26.9 Mg/ha	0.048	0.042	0.044	0.047	0.066
No gypsum	0.045	0.027	0.023	0.042	0.042

Dolomite (%)

	Soil depth (cm)				
	2	4	10	25	40
Glucose, 4.5 Mg/ha	1.559	1.368	1.260	0.942	0.542
No glucose	0.856	0.764	0.695	1.027	0.520

	Soil depth (cm)				
	2	4	10	25	40
Corn Residues, 13.4 Mg/ha	1.094	0.959	0.952	1.053	0.385
No residue	1.371	0.916	0.996	1.130	0.677

	Soil depth (cm)				
	2	4	10	25	40
Gypsum, 26.9 Mg/ha	1.109	0.895	0.655	1.164	1.262
No gypsum	1.059	0.860	0.407	1.159	0.962

Calcium carbonate equivalent (%)

	Soil depth (cm)				
	2	4	10	25	40
Glucose, 4.5 Mg/ha	1.786	1.537	1.415	1.076	0.619
No glucose	0.949	0.849	0.775	1.167	0.622

	Soil depth (cm)				
	2	4	10	25	40
Corn Residues, 13.4 Mg/ha	1.253	1.074	1.066	1.207	0.471
No residue	1.548	1.017	1.116	1.266	0.776

	Soil depth (cm)				
	2	4	10	25	40
Gypsum, 26.9 Mg/ha	1.251	1.013	0.755	1.309	1.435
No gypsum	1.194	0.960	0.464	1.319	1.086
